# Investigation of the performance of serological assays used for Lyme disease testing in Australia

**DOI:** 10.1371/journal.pone.0214402

**Published:** 2019-04-29

**Authors:** Susan J. Best, Marlene I. Tschaepe, Kim M. Wilson

**Affiliations:** National Serology Reference Laboratory, Division of St Vincent’s Institute of Medical Research, Melbourne, Victoria, Australia; University of Toledo College of Medicine and Life Sciences, UNITED STATES

## Abstract

Spirochaetes of the *Borrelia burgdorferi* sensu lato complex, which includes those that cause Lyme disease, have not been identified in Australia. Nevertheless, Australian patients exist, some of whom have not left the country, who have symptoms consistent with so-called “chronic Lyme disease”. Blood specimens from these individuals may be tested in Australian laboratories and in specialist laboratories outside Australia and sometimes conflicting results are obtained. Such discrepancies cause the patients to question the results from the Australian laboratories and seek assistance from the Australian Government in clarifying why the discrepancies occur. The aim of this study was to determine the level of agreement in results between commonly used *B*. *burgdorferi* serology assays in specimens of known status, and between results reported by different laboratories when they use the same serology assay. Five immunoassays and five immunoblots used in Australia and elsewhere were examined for the detection of IgG antibodies to *Borrelia burgdorferi* sensu lato. Predominantly, archived specimens previously tested for Lyme disease were used for the study and included 639 contributed by seven clinical laboratories located either in Australia or in areas endemic for Lyme disease. Also included were 308 prospectively collected Australian blood donor specimens. All clinical specimens were tested in all 10 assays whereas blood donor specimens were tested in all immunoassays and a subset was tested on immunoblots. With the exception of one immunoblot, the results between the assays agreed with each other in a known positive specimen population ≥ 77% of the time and in a known negative population, 88% of the time or greater. The test results obtained during the study were different from the participating laboratory’s less than 2% of the time when the same assay was used. These findings suggest that discordance in results between laboratories is more likely due to variation in algorithms or in the use of assays with different sensitivities or specificities rather than conflicting results being reported from the same assay in different laboratories. In the known negative population, specificities of the immunoassays ranged between 87.7% and 99.7%. In Australia’s low prevalence population, this would translate to a positive predictive value of < 4%.

## Introduction

Lyme disease is caused by specific spirochaetes of the *Borrelia burgdorferi* sensu lato *(*sl*)* complex. It is transmitted to humans through bites by infected ticks typically of the *Ixodes* species. Lyme disease is prevalent in specific parts of Europe, North America and North Asia. Several studies examining Australian ticks for the presence of *B*. *burgdorferi* sl, or their competence to transmit the organism, have not yielded positive results [1, 2]. Similarly, research recently published could not identify organisms of the *B*. *burgdorferi* sl complex in Australian ticks [3]. However, this research did detect a *Borrelia* species from a proposed new clade of *Borrelia* in a single *Ixodes holocyclus* tick, causing the author to suggest that this finding may warrant further investigation in the context of a locally acquired “Lyme–like illness” in Australia.

Significant controversy surrounds the question of whether Lyme disease can be locally acquired in Australia. A group of patients exists, many of whom have not travelled outside Australia, who exhibit symptoms described by advocates as “chronic Lyme disease”. These patients have produced negative results using commercially available serological assays in some Australian laboratories but have been given positive test results for Lyme disease from other laboratories both within and outside Australia [4]. These positive results are not necessarily obtained in commercial serology assays, with several laboratories offering a suite of tests that use different technologies, (for example Borrelia enzyme linked immunospot (ELiSpot), CD57+ count, RT PCR) from which a positive overall result is interpreted. The use of these different technologies has been challenged as having not been sufficiently evaluated for their usefulness in diagnosis and being difficult to standardise [5, 6]. This disagreement in results between laboratories has raised questions about the serology assays used for testing specimens from Australian individuals in medical testing laboratories in Australia and overseas. Without evidence of endemic Lyme disease in Australia, the Royal College of Pathologists of Australasia caution that many positive serology results are false based on the low predictive value of a positive result in Australia’s population, which has a low prevalence of Lyme disease [7].

Terminology adds to the confusion. “Lyme disease”, “Lyme-like illness” and “Chronic Lyme disease” are all terms used to refer to a range of non-specific symptoms suffered by these patients. Patient support groups consider that other co-infecting tick-borne pathogens may contribute to these symptoms [8]. However, when a laboratory receives a request to test a specimen for Lyme disease, the serology assays used detect only antibodies to *B*. *burgdorferi* sl. species. The report of a Senate inquiry conducted by the Australian Government has made many recommendations that, once implemented, will seek to develop a standard, “multi-disciplinary approach” to tick-borne illness in Australia and undertake research aimed at identifying pathogens that may be contributing [8].

The study reported here was funded by the Australian Government Department of Health, which contracted the National Serology Reference Laboratory, Australia (NRL) to undertake the investigation. The aim was to examine the agreement between the results of different serology assays used by Australian and overseas laboratories to test specimens from Australian individuals for Lyme disease. The study was designed to determine the ability of the tests to detect IgG antibodies to *B*. *burgdorferi* sl and not to other *Borrelia* species. The study included assays widely used in Australia and overseas to test specimens that had been previously tested for Lyme disease and that were collected from Lyme endemic and non-endemic countries.

A number of clinical laboratories that conduct serological testing for Lyme disease were approached to participate in this study by providing serum specimens of known provenance that were banked in their archives. The laboratories were located both within and outside Australia and, irrespective of their location, were likely to have tested specimens from Australian individuals. If known, specimens were requested to have been collected from individuals whose symptoms, where relevant, had passed the acute phase. Hence, *B*. *burgdorferi* IgG antibodies were expected to be present in the sera provided that had been collected from true cases of Lyme disease. In our study, the specimens were retested in the commercially available *Borrelia burgdorferi* IgG assays that had been used by the clinical laboratories. Taking this approach allowed us to assess the reproducibility of the assays (testing the same specimen in the same assay at a different laboratory) and to examine the agreement between the results of different assays on the same specimen. In addition, specimens were collected prospectively from consenting Australian blood donors for inclusion in the study.

## Materials and methods

### Specimens

Seven clinical laboratories agreed to provide archived specimens to the study, three in Australia, one in the United Kingdom, two in Germany and one in the United States. These laboratories were able to provide serum specimens that had been stored at ≤ –20°C since collection, and that had a minimum volume of 800μL and test results from one or more IgG assays for B. burgdorferi sl. Further, these laboratories were willing to provide, where available, pertinent information for the purposes of the study. Information requested included, date of collection, relevant clinical information, specimen storage temperature, test results in IgG assays for B. burgdorferi and interpretation of the results. Laboratories were asked to provide specimens that they had deemed IgG positive, negative or equivocal / indeterminate for the study. The non-Australian laboratories were approached to provide specimens for the study because they were located in Lyme endemic areas and some were likely to have tested specimens from Australia. In total, 639 specimens of the required volume were provided by the seven clinical laboratories. The breakdown of these specimens is shown in Table 1.

**Table 1:** Number and *B*. *burgdorferi* IgG antibody status of specimens provided by participating laboratories.

Laboratory ID	Laboratory location	*B*. *burgdorferi* IgG antibody status		
		Positive	Negative	Equivocal / Indeterminate
A	Australia	4	53	0
B	Australia	2	7	9
C	Australia	5	58	14
D	EU country*	100	100	50
E	EU country	56	33	0
F	EU country	57	31	0
G	United States	24	27	9
Blood Service	Australia	0	308	0

* Each of the countries designated “EU country” was part of the European Union as at January 2019.

Australian blood donor specimens were collected prospectively for the study by the Australian Red Cross Blood Service. The specimens were collected from sequential consenting Tasmanian blood donors presenting to donate blood in Hobart, Devonport and Burnie. Blood donors from these regions were sought because ticks are less common in north western and southern Tasmania than on the east coast of mainland Australia [9]. Consenting donors were asked if they had travelled outside Australia and only those who had not were recruited for the study. Otherwise, the specimens were anonymised; demographic information was not recorded. Whole blood was collected into serum separator tubes, which were centrifuged within 48 hours of collection then stored at 4°C for up to five days before removal of serum into aliquots and freezing. Ethics approval was obtained for the collection of the blood donor specimens under St Vincent’s Hospital Melbourne Human Research Ethics Committee Protocol number 079/05.

A range of result combinations was provided on the specimens from different participating clinical laboratories. Two laboratories provided results on a single *B burgdorferi* IgG test only, in both cases an immunoblot. Another provided results on both an immunoassay and an immunoblot on some specimens, and on immunoblot only for the remainder. This laboratory indicated that an immunoblot was the sole test performed only when so requested by a clinician. The remaining four clinical laboratories provided results on two or more *B burgdorferi* IgG assays. For the purposes of Table 1, “Positive” was assigned to a participating laboratory’s specimens when their single assay or all their IgG assays were positive; “Negative” when their single assay or results interpreted using their 2-tier algorithm were negative; and “Equivocal / Indeterminate” when this was the status assigned by the laboratory or when this was the interpreted result of a single *B burgdorferi* immunoassay or immunoblot. Laboratory G used in-house IgG assays and gave an overall interpretation for each specimen provided, which was used in Table 1.

The blood donor specimens had not been tested for *B burgdorferi* previously. For the purposes of assigning a result status they were classified as negative.

### Serological assays

The assays chosen to be included in the study were those that were currently used for serology testing for anti*-B*. *burgdorferi* IgG or had been used in the previous three years by the participating laboratories. The assays are shown in Table 2.

**Table 2:** Name, manufacturer and preparation of antigens used in the assays included in the study.

Assay Name (Abbreviation)	Antigen type	Manufacturer
**Immunoassays**		
NovaTec NovaLisa *Borrelia burgdorferi* IgG-ELISA (Novatec Novalisa ELISA)	Recombinant	Novatec Immundiagnostica, Dietzenbach, Germany
DiaSorin LIAISON *Borrelia* IgG Chemiluminescent immunoassay (DiaSorin LIAISON CLIA)	Recombinant	DiaSorin S.p.A., Italy
Trinity Biotech *B*. *burgdorferi* ELISA (IgM/IgG) Test System (Trinity Biotech ELISA)	Native	MarDX Diagnostics Inc* California, United States
EUROIMMUN Anti-*Borrelia* Select ELISA (Euroimmun ELISA)	Recombinant	EUROIMMUN Medizinische Labordiagnostika Germany
Immunetics C6 Lyme ELISA (Immunetics C6 ELISA)	Peptide	Immunetics Inc, Boston USA
**Immunoblots**		
Viramed *Borrelia* ViraStripe IgG Immunoblot (Viramed ViraStripe)	Native / recombinant	Viramed Biotech, Planegg, Germany
EUROIMMUN Anti-*Borrelia* EUROLINE-RN-AT (IgG) Immunoblot (Euroimmun Euroline)	Recombinant	EUROIMMUN Medizinische Labordiagnostika Germany
Mikrogen recomLine *Borrelia* IgG Immunoblot (Mikrogen recomLine)	Recombinant	Mikrogen GmbH Nueried Germany
Trinity Biotech EU Lyme +VlsE IgG Wstern Blot (Trinity Biotech)	Native / recombinant	MarDX Diagnostics Inc* California, United States
Seramun SeraSpot Anti-*Borrelia*-10 IgG (Seramun SeraSpot)	Recombinant	Seramun Diagnostica, Heidesee, Germany

* MarDx Diagnostics is a subsidiary of Trinity Biotech, Bray, Ireland

Of the five assays referred to as immunoassays, four were microplate-based enzyme immunoassays and one was an instrument based chemiluminescent immunoassay (DiaSorin LIAISON CLIA). Of the five assays referred to as immunoblots, four presented their antigens on strips and the last was a spot immunoassay (SIA), which presented *B*. *burgdorferi* antigens as spots on the base of microtitre wells (Seramun SeraSpot). The results of the SIA are read and interpreted using the same approach to interpretation as immunoblots. Therefore, the SIA was grouped with the immunoblots for the purposes of this study. A single lot number of each of the assays was used for the study.

We chose to test the specimens for IgG antibodies only for a number of reasons. To fulfil the aims of the study, we wished to include as close to a complete range of the different manufacturers’ assays that were used by the participating laboratories as possible. If we had included dedicated IgM assays as well as those for IgG, the volume of archived serum would not have been available and consequently the number of different manufacturers’ assays would have needed to be restricted. Although there are reports of *B*. *burgdorferi* sl IgM antibodies being present in late stage Lyme disease [10,11], concern around false positive IgM results is reported [12, 13]. Further, the debate in Australia is not focused on individuals with symptoms consistent with acute Lyme disease. It has been shown that Australian laboratories can detect antibodies in acute Lyme disease once the interval after infection required for antibody response has elapsed [14]. Rather, the debate is focused on individuals whose symptoms are non-specific and described as “chronic Lyme disease”.

### Testing

All the study specimens provided by clinical laboratories were tested in all 10 assays as opposed to determining a specimen’s result according to a two-tier algorithm. The reason for this was two-fold. First, two of the clinical laboratories used an immunoblot as the first, and in some cases the only test. Second, the instructions for use for only three of the immunoblots (Trinity Biotech, Mikrogen recomLine, Seramun SeraSpot) specified that they should be used only on specimens that were reactive on an ELISA or IFA. Therefore, it was considered beneficial to discover how the immunoblots would perform if they were used as a first and/or only test.

Of the blood donor specimens, all 308 were tested in all five immunoassays and 132 were also tested on all the immunoblots. Although it is not recommended to test negative specimens in immunoblots, these 132 blood donor specimens were tested for the same reasons as already discussed. The 132 specimens consisted of eighty-seven that had given reactivity at least once in any of the immunoassays, and 45 chosen sequentially from the remainder.

All testing for the study was performed by NRL according to the assay manufacturers’ instructions for use. Specimens that gave grey zone / equivocal / borderline results were retested when the instructions for the relevant assay specified to do so. In these cases, the result for the repeat test was the one recorded.

Of the five immunoblots, dedicated scanners and software for reading and interpreting the results were used for the Euroimmun Euroline and for the Seramun SeraSpot. The remaining three immunoblots were read by eye. Methods to maximise consistency in reading by eye included having two medical scientists read and record reactivity for each strip independently, followed by comparison of results scored by each. Occasionally the two scientists could not agree and in these cases a third was consulted.

## Result interpretation

Results of all the assays were interpreted according to the relevant manufacturer’s instructions.

Of the five immunoassays included in the study, all had criteria for grey zone / equivocal / borderline result interpretation. Two (DiaSorin LIAISON CLIA and Immunetics C6 ELISA) suggested a single repeat test be performed on the same specimen in the case of a grey zone result; the remainder suggested another specimen be collected in different numbers of days or weeks. Only one of the immunoassays (Trinity Biotech ELISA) specified that an equivocal or reactive result should be confirmed by immunoblot before the result is reported.

Four of the five immunoblots had criteria for indeterminate / equivocal / borderline result interpretation; the Euroimmun Euroline gave criteria for positive or negative interpretation only. One (Trinity Biotech) gave two options for result interpretation: “Interpretive Criteria for Europe excluding FDR Germany” and “Interpretive Criteria for FDR Germany”, the latter being less stringent. Those for “Europe excluding FDR Germany” were used to interpret the results from the study specimens.

Whether read by eye or digitally, in a microwell or on a strip, all the immunoblots used the principle of comparing the intensity of a band, or spot in case of SeraSpot, with that of a cut-off control band (spot) to assign reactivity. If the intensity of the test band (spot) was equal to or greater than the cut-off control band (spot), the test band (spot) was considered reactive.

When reporting the overall interpretation of results, three of the five immunoblots reported VlsE reactivity alone as either indeterminate (borderline) or positive. Table 3 shows the number of bands (spots) required to be reactive to assign indeterminate or positive overall interpretation. The information in the table provides an overview of the different immunoblots’ interpretation criteria. The specific details of each are difficult to summarise because the different immunoblots use different scoring systems (for example one assigns a point value to each of the bands) and some assign increased significance to the presence of some bands (e.g. VlsE).

**Table 3:** Interpretation criteria for each of the immunoblots included in the study.

	Number of bands (spots) required to show reactivity ≥ cut-off control band (spot)		
Immunoblot	Negative	Borderline (Indeterminate)	Positive
Trinity Biotech (Excl FDR Germany)	< 2	2	≥ 3
Viramed ViraStripe	≤ 1 (except VlsE)	1 (VlsE)	≥ 2
Mikrogen recomLine	≤ 1	2 (incl p41)	≥ 2
Euroimmun Euroline	≤ 1	Not applicable	1 (VlsE) or ≥ 2
Seramun SeraSpot	≤ 1 (except VlsE)	1 (VlsE)	≥ 2

### Data analysis

#### Delta values

Delta (δ) values were determined for all of the immunoassays except the DiaSorin LIAISON CLIA. The delta (δ) of an immunoassay is a measure of the distance of the mean sample to cut-off ratio (S/CO) of a positive or negative specimen population from the cut-off of the assay, measured in standard deviations (SD) [15]. Briefly, the S/COs in each of the relevant immunoassays for each of the specimens in the known positive and known negative populations were log_10_ transformed and the mean and SD determined. To determine the positive and negative δ values, the mean log_10_ transformed S/CO of the relevant specimen population’s results was divided by the SD of the log_10_ transformed results. Assuming a normally distributed population, a δ value of 1 (-1) for an immunoassay would indicate that the mean of the assay’s log_10_ transformed positive (negative) results would be one SD from the cut-off. Similarly, when the δ value is 2 (-2) or 3 (-3), those assays’ results would be 2 and 3 SD from the cut-off respectively. Therefore, ideally, an assay’s δ value will be ≥ 3 to ensure that all the results in a population are sufficiently removed from the cut-off of the assay. Consequently, the lower the δ value the greater the propensity of an immunoassay to give false results. A δ value could not be determined for the DiaSorin LIAISON CLIA because the results are automatically calculated by the LIAISON XL instrument and expressed as arbitrary units.

#### Sensitivity and specificity

The results were separated into two working datasets, one that included the results of the clinical specimens and another that included those of the blood donor specimens. The data were analysed using two different approaches.

#### Sensitivity in known positive specimens

The specimens’ results used for sensitivity analysis were the 100 specimens with positive antibody status provided by Laboratory D. These were chosen because the specimens were collected from individuals that had relevant clinical history, had originated from areas where Lyme disease is prevalent and the positive result was obtained using a two-tier algorithm.

The sensitivity for each of the study assays and positive δ values for the four immunoassays for which it could be determined were estimated using results from this panel of specimens.

#### Specificity in known negative specimens

The blood donor specimens’ results were used for specificity analysis (n = 308). All blood donor specimens were considered *B*. *burgdorferi* IgG negative given the history of no travel outside Australia and the presumption that the donors were free of symptoms of Lyme disease because blood donors are required to feel well and meet a number of health screening requirements in order to donate.

The specificity for each of the study assays and negative δ values for the four immunoassays for which it could be determined were estimated using results from this panel of specimens.

When calculating sensitivity or specificity, equivocal / borderline / indeterminate results were considered negative when estimating sensitivity and positive when estimating specificity. Using this approach, conservative estimates of sensitivity and specificity were calculated.

#### Positive and negative agreement when the *B*. *burgdorferi* IgG status was presumed

In those specimens that were not allocated to the known positive or negative specimen panels, an IgG antibody status was presumed based on the specimens’ reactivity in the 10 assays. A presumed status of positive was allocated to a specimen if it was positive in seven of the 10 assays used in the study. Similarly, a presumed status of negative was allocated when a specimen was negative in seven of the 10 assays.

In this article, “positive agreement” (or “negative agreement”) are used instead of “sensitivity (specificity)” to describe the performance of the assays in the presumed positive (negative) specimen panels respectively, as the status of these specimens was assigned based on serological consensus only, in contrast to those described as “known positive (negative)”. The estimation of positive and negative agreement for specimens where the status was “presumed” was conducted separately from those where the status was “known”.

#### Agreement between clinical laboratories’ and study results

Clinical laboratories that provided specimens to the study also provided the results obtained in the serological assays that had been used. Where possible, these results were compared with the results obtained in the study using the same assay.

## Results

### Sensitivity and specificity

Information provided by the participating laboratories always included the specimens’ dates of collection, storage temperatures and IgG results. Otherwise, information such as clinical and travel history, gender and age was inconsistently provided.

Table 4 shows the assays’ sensitivities and specificities estimated from the study testing in the known positive and known negative specimens respectively. Also included in Table 4 are the δ values for those assays for which it could be calculated, the 95% confidence intervals around the sensitivity and specificity estimates, and the number of positive, negative and equivocal results obtained using each assay.

**Table 4:** The sensitivity, specificity, 95% confidence intervals (CI) and delta values of assays in known *B*. *burgdorferi* IgG positive and negative specimen panels respectively. Equivocal results are considered negative for sensitivity and positive for specificity estimations.

	Known positive panel (specimens = 100)						Known negative panel						
Assay	Pos (n)	Neg (n)	Equivocal (n)	Sensitivity (%)	95% CI	δ value	Specimens (n)	Pos (n)	Neg (n)	Equivocal (n)	Specificity (%)	95% CI	δ value
Novatec Novalisa ELISA	94	5	1	94	87–98	1.91	308	1	307	0	99.7	98–100	-2.91
DiaSorin Liaison CLIA	95	4	1	95	89–98	N/A		9	297	2	96.4	94–98	N/A
Trinity Biotech ELISA	80	13	7	80	71–87	1.2		12	282	14	91.6	88–94	-1.31
Euroimmun ELISA	78	14	8	78	69–86	0.97		0	307	1	99.7	98–100	-2.99
Immunetics C6 ELISA	100	0	0	100^a^	96–100	4.03		33	270	5	87.7	83–91	-1.06
Viramed ViraStripe IB	89	2	9	89^a^	81–94	N/A	132	0	131	1	99.2	96–100	N/A
Euroimmun Euroline IB	99	1	N/A	99	95–100	N/A	135	7	128	N/A	94.8	90–98	N/A
Trinity Biotech IB	33	61	6	33	24–43	N/A	132	0	132	0	100	97–100	N/A
Mikrogen recomLine IB	77	9	14	77	67–85	N/A		1	130	1	98.5	95–100	N/A
Seramun SeraSpot	87	8	5	87	78–93	N/A		6	125	1	94.7	89–98	N/A

^a^The Immunetics C6 ELISA and the Viramed ViraStripe immunoblot (IB) were used by Laboratory D to assign positive status to the specimens in the known positive specimen panel. N/A = not applicable

The estimates of sensitivity in the immunoassays ranged from 78% to 100% (Table 4). (Note that the estimate of 100% for the Immunetics C6 ELISA was not unexpected because this assay was used by Laboratory D as the first assay in its two-tier algorithm, a reactive result in which was required for a specimen to be included in the known positive specimen panel. In our testing we also obtained positive results in all 100 specimens in the Immunetics C6 ELISA). The δ values were 0.97 (Euroimmun ELISA), 1.2 (Trinity Biotech ELISA), 1.9 (Novatec Novalisa) and 4.03 (Immunetics C6 ELISA). The Trinity Biotech ELISA and the Euroimmun ELISA gave seven and eight equivocal results respectively, while the remaining two immunoassays gave one equivocal result each.

The estimates of sensitivity in the immunoblots ranged from 33% to 99%. In our testing, the Viramed ViraStripe gave positive results in 89 and equivocal results in 9 of the 100 known positive specimens. Laboratory D had found positive results in all 100 of these specimens using this assay. The results in the 9 specimens that gave equivocal results when tested for the study were compared with those obtained by Laboratory D. In every case, Laboratory D had considered one additional band as having reactivity equal to or greater than the cut-off control band whereas our evaluation had considered the reactivity of the same band less than the cut-off control band. Laboratory D used a scanner to interpret the immunoblot results whereas results were interpreted by eye in our study.

The Trinity Biotech immunoblot showed poor sensitivity of 33% in the known positive specimen panel. The sensitivity of the remaining immunoblots ranged from 77–99%.

Of the 308 Australian blood donor specimens, 87 showed initial equivocal or positive reactivity in one or more assays (Table 4). The instructions for the DiaSorin LIAISON CLIA and Immunetics C6 ELISA recommended that specimens with equivocal results were retested on the same specimen. Of 14 specimens initially equivocal on the Immunetics C6 ELISA, 11 were negative on retesting, two remained equivocal and one became positive. In addition, 10 specimens initially positive on the Immunetics C6 ELISA were retested once. These were specimens that had shown reactivity on at least one other assay in addition to the C6 ELISA. Of these 10, seven remained positive and three became equivocal on retesting. Hence, all 10 were considered falsely reactive for the purposes of specificity estimation. Of four specimens originally equivocal on the DiaSorin LIAISON CLIA two remained equivocal and two returned positive results on retesting. After these adjustments, for the purpose of analysis, 78 specimens in the known negative panel were recorded as reactive in one or more assays.

The estimates of specificity in the immunoassays in the known negative population ranged from 87.7%–99.7%. The δ values of the Novatec Novalisa ELISA and the Euroimmun ELISA were both greater than 2.9 while the δ values for the remaining two assays for which the statistic could be calculated were ≤ 1.3. The Trinity Biotech ELISA gave 14 (4.5%) equivocal results in the known negative specimen panel. Otherwise, the number of equivocal results was ≤ 5 in all the other assays with two (Novatec Novalisa ELISA and Trinity Biotech immunoblot) giving no equivocal results at all. Despite immunoblots not typically being used for testing negative specimens, the specificity estimates for the immunoblots in the known negative specimen panel were all greater than 94.5%.

Of the 78 blood donor specimens that were equivocal or reactive, 63 were reactive in only one of 10 assays. The remaining 15 specimens were reactive in two or more assays: 14/78 were reactive or equivocal in two and 1/78 was reactive in one and equivocal in two more of the 10 assays. Of the 78, the rate of reactivity was highest in the Immunetics C6 ELISA with 38 of the 78 being equivocal or reactive in one or more assays. The Trinity Biotech ELISA showed reactivity in 26 of the 78 specimens.

### Positive and negative agreement when the *B*. *burgdorferi* IgG status was presumed

After allocating 100 specimens to the known positive panel, there were 95 specimens of the remaining 539 that gave positive results in seven of the 10 assays. These formed the presumed antibody positive specimen panel.

Similarly, of the 539 there were 405 specimens in which seven of the 10 assays were negative. These formed the presumed antibody negative specimen panel.

The study results of 39 specimens were not analysed because a status could not be assigned to them; none of these 39 specimens was positive or negative in seven of 10 assays.

The positive agreement of the immunoassays in the presumed antibody positive specimens ranged from 73% to 100%. Two of the five immunoassays (Novatec Novalisa ELISA and Immunetics C6 ELISA) gave positive results in 100% of the specimens and four of the five gave positive results in ≥ 94% of specimens in this panel (Table 5).

**Table 5:** The positive and negative agreement of the study assays in specimens with presumed *B*. *burgdorferi* positive and negative IgG status respectively. Equivocal results obtained in the study are considered negative for estimation of positive agreement and positive for estimation of negative agreement.

	Presumed antibody status positive panel (n = 95)				Presumed antibody status negative panel (n = 405)				
Assay	Positive (n)	Negative (n)	Equivocal (n)	Positive agreement (%)	Positive (n)	Negative (n)	Equivocal (n)	Negative agreement (%)	Adjusted negative agreement (%)^a^
Novatec Novalisa ELISA	95	0	0	100	14	385	6	95.1	94.9
DiaSorin Liaison CLIA	94	1	0	99	23	372	10	91.9	92.5
Trinity Biotech ELISA	69	22	4	73	24	360	21	88.9	89.5
Euroimmun ELISA	89	4	2	94	4	397	4	98.0	97.9
Immunetics C6 ELISA	95	0	0	100	78	322	5	79.5	90.1
Viramed ViraStripe IB	88	2	5	93	6	387	12	95.6	95.2
Euroimmun Euroline IB	95	0	N/A	100	35	370	N/A	91.4	91.3
Trinity Biotech IB	63	17	15	66	0	405	0	100	100
Mikrogen recomLine IB	88	3	4	93	0	397	8	98.0	98.2
Seramun SeraSpot	95	0	0	100	12	390	3	96.3	96.1

^a^ Percent agreement adjusted after removal of specimens contributed as “equivocal / indeterminate” by participating laboratories (adjusted n = 332)

Results in the presumed positive specimen panel implied better performance of the immunoblots (Table 5) when compared with results in the known positive specimen panel. In the Trinity Biotech immunoblot, positive agreement in the presumed positive panel was 66% compared with 33% in the known positive panel; in the Mikrogen recomLine 93% compared with 77% and in the Seramun SeraSpot, 100% compared with 87%. Positive agreements in the other two immunoblots were within 5% of each other irrespective of positive specimen panel. The positive agreement of the Trinity Biotech immunoblot remained low when compared with the other immunoblots, which all agreed with the presumed positive status of > 90% of the specimens in this panel.

The negative agreement in all the assays except the Immunetics C6 ELISA was ≥ 89% in the presumed negative specimen panel. In the Immunetics C6 ELISA, 79.5% of results were negative in this panel with a corresponding δ value of –0.74. However, this apparently lower specificity of the C6 ELISA in this panel may be biased by the specimens the panel comprises. Table 1 shows the breakdown of specimens supplied by collaborating laboratories. It can be seen from Table 1 that, in addition to 100 specimens it deemed *B*. *burgdorferi* IgG positive, Laboratory D contributed another 50 specimens that it had deemed equivocal / indeterminate. The equivocal status assigned by Laboratory D was based on reactivity in the Immunetics C6 ELISA that was not confirmed by immunoblot. Of these 50, 45 were negative in seven or more of the assays in the study testing and therefore were allocated to the presumed negative panel. Similarly, Laboratories B, C and G had contributed 5, 14 and 9 specimens respectively that they had deemed equivocal / indeterminate and that had been allocated to the study’s presumed negative panel based on their reactivity in the study testing. If we remove these equivocal / indeterminate specimens from the panel and recalculate the negative agreement of all the assays, the effect is small (≤ 0.6%) for all except the Immunetics C6 ELISA. For this assay, the negative agreement changes from 79.5% to 90.1% when the equivocal / indeterminate specimens are removed.

A similar adjustment is not required for any other specimen panel in the study. Of the 82 specimens contributed as “equivocal / indeterminate”, 73 were allocated to the presumed negative panel by virtue of their reactivity in the study testing. The remaining nine are included in the 39 specimens with results in the study that could not be analysed because they were not positive or negative in seven of 10 assays.

### Agreement between clinical laboratories’ and study results

Of the 639 specimens contributed by the clinical laboratories results from 502 were able to be compared with the results obtained in this study using the same assay. Results from 137 samples contributed by two of the clinical laboratories could not be compared because the laboratory had either used in house tests or had reported results from a modification to the procedure stated in the relevant assay’s instructions. A total of 724 results were provided by the clinical laboratories for the 502 specimens across eight of the assays. Comparing the corresponding results that were obtained in the study, 14 (1.9%) were discordant; in other words, the result was negative by the clinical laboratory but positive in the study, or vice versa. An additional 26 were equivocal in either the clinical laboratory or the study when the counterpart result was either negative or positive (Table 6).

**Table 6:** Agreement between specimens’ results obtained in the clinical laboratory and results obtained in this study in the same assay.

Assay	Total results compared (n)	Discordant results^a^ (n)	Equivocal results^b^ (n)
Novatec Novalisa ELISA	2	0	1
DiaSorin LIAISON CLIA	73	0	4
Euroimmun ELISA	49	0	4
Immunetics C6 ELISA	250	4	2
Viramed ViraStripe IB	150	2	14
Euroimmun Euroline IB	101	6	0
Trinity Biotech IB	11	2	2
Seramun SeraSpot	88	0	0
TOTAL	724	14	26

^a^ Discordant: those results negative by the clinical laboratory but positive in the study, or vice versa.

^b^ Equivocal: those results equivocal in the clinical laboratory and positive or negative in this study or vice versa (positive or negative in the clinical laboratory and equivocal in this study).

## Discussion

A total of 771 clinical and blood donor specimens have been tested in five immunoassays and five immunoblots that detect IgG antibodies to spirochaetes of the *B*. *burgdorferi* sl complex; a further 176 blood donor specimens were tested in the five immunoassays only. Positive, negative and equivocal specimens were contributed by participating laboratories located in both Lyme-endemic and non-endemic areas. The main objectives of the testing were to examine the performance of the 10 assays in specimens originating from both within and outside Australia, and from this to infer whether different serology results in these assays were obtained. While using a classical two-tier algorithm for Lyme disease testing may be considered a gold standard approach [16], differences in the antigens used and quality of the serological assays available for this testing mean that different final interpretations can be obtained when different assays are used for each of the tiers [17]. Therefore alternative approaches to assigning positive and negative status to the specimens in this study has resulted in separate sensitivity and specificity analyses in specimens known or presumed to be positive or negative respectively.

In the known positive specimens tested as part of this study, the Novatec Novalisa ELISA and DiaSorin LIAISON CLIA gave sensitivities of 94% (δ value 1.91) and 95% (δ value not applicable) respectively. On the other hand the Trinity Biotech ELISA and the Euroimmun ELISA gave poorer sensitivities of 80% and 78% respectively and gave more equivocal results (7 and 8 respectively). These differences in performance are borne out by the δ values in these last two immunoassays which were 1.2 and 0.97 respectively. This means that the mean results in this positive population using these assays were only one standard deviation from the cut-off of the assay and therefore the higher proportion of negative and equivocal results is not unexpected.

All the immunoassays except the Trinity Biotech ELISA used recombinant or peptide antigens on the solid phase. The Trinity Biotech ELISA used sonicated whole cell antigens. Properly-constructed and targeted recombinant and peptide antigens generally lead to assays that are more sensitive and specific because the antigens can be effectively purified or synthetically manufactured, and cross reactive regions can be omitted [18, 19, 20]. Further, the antigens in the Trinity Biotech ELISA were derived only from the *B burgdorferi* sensu stricto strain, which, although present in both Europe and North America, is more often associated with Lyme disease in North America [21]. This may have affected this assay’s sensitivity in this study, given that, of the known positive (n = 100) and presumed positive (n = 95) specimen panels, only 14 had originated in North America. Of the remaining 181, 100 were assumed and 77 were known to have originated from the UK or a western, central or eastern European country.

The proportion of negative results given by the immunoassays was > 91% in the known negative specimen panel. The exception was the Immunetics C6 ELISA, giving a specificity of 87.7%. The negative δ value of –1.06 for this assay predicted the higher false positive rate seen with the Immunetics C6 ELISA.

Seventy-eight of 308 Tasmanian blood donor specimens were equivocal or reactive in one or more of the study assays; 63 of these were reactive in one assay only and of these, 27 and 22 were reactive in the Immunetics C6 or the Trinity Biotech ELISAs respectively. Considering that the known negative panel only contained specimens from Australia, if these immunoassays were to be used in the low prevalence Australian population, the positive predictive value (PPV) of their unconfirmed results would be very low. There is no known prevalence of Lyme disease in Australia. If we assumed a prevalence of 0.1% amongst patients being tested for Lyme disease, allowing for travellers to and from endemic areas, the predictive value of a positive result from any of the immunoassays would be less than 4%. Hence a two-tier approach in Australia is recommended to avoid these false positive results [7].

Over the last decade, modified testing algorithms, in which the second tier immunoblot used in the classical approach is replaced with a second ELISA [22, 23, 24, 25] or where a C6 ELISA is used alone [26] have been evaluated. The evaluations sought to determine whether the modifications would increase sensitivity in early infection, reduce the subjectivity introduced by immunoblot testing but maintain specificity. The evaluations acknowledge the low sensitivity in early infection of classical two-tier testing, using CDC immunoblot interpretation criteria, and recognise the need for improved diagnostic accuracy of laboratory testing for Lyme disease. Nevertheless, some of these reports recommend maintaining the current CDC recommendations [24, 26]. Slight reductions in specificity using a C6 ELISA alone compared with the classical two-tier algorithm using both IgG and IgM immunoblots were reported [22, 26]; in one low prevalence population, this small reduction in specificity led to a substantial decrease in the positive predictive value of a C6 ELISA alone result [22]. All of these evaluations included algorithms that incorporated the C6 ELISA and reported enhanced sensitivity when the C6 ELISA was used especially in early infection. In our study, the 100 specimens that made up the known positive specimen panel were contributed by Laboratory D, who used a two-tier algorithm consisting of the Immunetics C6 ELISA and the Viramed ViraStripe immunoblot, both tests needing to be positive for inclusion in the panel. The study testing also found these 100 specimens positive in the Immunetics C6 ELISA. In the remaining ELISAs, sensitivities in this panel of 95% (DiaSorin LIAISON CLIA), 94% (Novatec NovaLisa), 80% (Trinity Biotech ELISA) and 78% (Euroimmun ELISA) support the reports of increased sensitivity in the C6 ELISA. In the presumed positive specimen panel, none of the specimens had been screened in the Immunetics C6 ELISA by the contributing laboratory. Nevertheless, the assay was positive in all 95 specimens, a further indication of its high sensitivity. One other immunoassay (Novatec Novalisa ELISA) and two immunoblots (Euroimmun and Seramun SeraSpot) were also positive in all 95 specimens.

If algorithms containing two or three immunoassays were used instead of including immunoblots, it would be important to consider not only (i) the candidate immunoassays’ sensitivity and specificity, and (ii) which immunoassay would be used first in the algorithm but also (iii) to validate that the immunoassays combined did not give falsely reactive results in the same specimens. In the “presumed” antibody negative population included in this study, 34 of 405 specimens gave equivocal or positive reactivity in two (33) or three (1) immunoassays. This reactivity, which was deemed false according to the study parameters, was most frequently observed when the Immunetics C6 and the Trinity Biotech ELISAs were used in combination (14 specimens) followed by the combination of the Immunetics C6 ELISA and the DiaSorin LIAISON CLIA (10 specimens). No assay combination showed common false reactivity in ≥ 3 assays.

Fifteen blood donor specimens were reactive or equivocal in two (14) or three (1) of the study assays. Four of these gave results that fulfil the criteria for a positive result in a two-tier algorithm. The positive result in all four immunoblots was conferred by reactivity to two or in one case three antigens. The manufacturer’s instructions for all of the assays indicated that they should be used to test people with symptoms of Lyme disease. Therefore, the positive predictive value of any positive results in the known negative population used in this study would be very low [27, 28].

Several of the assays used in this study have been compared previously [14, 29]. Dickeson et al compared the NovaLisa, Trinity Biotech and Euroimmun ELISAs and the Trinity Biotech and Euroimmun immunoblots. They used a similar approach to ours in assigning status by consensus. A key difference between the studies was the source of the specimens. All specimens that Dickeson et al used, were sourced from Australia or New Zealand whereas, in the present study, only 24% of the clinical specimens were collected in Australia, the remainder having been collected in Lyme endemic areas. In the assays in common, similar findings between their study and ours were the specificities of 87% or greater in their negative specimen subset (n = 23) compared with specificities of 87.7% in our known negative panel and 79.5% or 90.1% (including or excluding C6ELISA equivocal / indeterminate specimens respectively; Table 5) in the presumed negative specimen panel. There was also good negative agreement between the immunoblots in common between the studies, and positive and negative agreements between the Novatec Novalisa and Euroimmun ELISAs. An exception was the Trinity Biotech immunoblot. Dickeson et al found that this assay gave positive results in 74% of the total positive specimens in their study (n = 66), while we found it gave positive results in only 33% of our known positive and 66% of our presumed positive specimens. It was not clear from the Dickeson et al publication which of the two interpretation criteria for the Trinity Biotech immunoblot they had used to interpret their results from this assay. If they used the less stringent criteria, it could account in part for the different findings. The assays in common between our study and that of Busson et al were the DiaSorin LIAISON CLIA, and the Euroimmun, Mikrogen recomLine and Viramed ViraStripe immunoblots. Percentage agreements with assigned status between the studies in both negative and positive specimen panels, in the assays in common, were within 7% of each other. The better correlation between our results and those of Busson et al compared with those of Dickeson et al may be due to greater similarity in the regions in which the specimens were collected.

During the study, of the 2313 immunoblots that were read by eye, approximately 7% required consultation of a third reader. We note that this approach to reading immunoblots was also undertaken by CDC when establishing a serum repository for evaluation of Lyme disease serology assays [30]. Differences in subjective determination of reactivity are not uncommon; in the case of the *B*. *burgdorferi* immunoblots, the impact of the subjective interpretation is exacerbated by the significant change in result brought about by the presence of a single additional band (spot). Reactivity to two proteins on most of the immunoblots is sufficient to confer a positive result, according the manufacturers’ instructions.

Thirty-three and 66% of results were positive using the Trinity Biotech immunoblot in the known positive and presumed positive specimen panels respectively. This immunoblot includes a purified VlsE protein of *B*. *burgdorferi*, which should increase its sensitivity in early infection. Unlike the Trinity Biotech ELISA, the immunoblot also contains native antigens derived from *B*. *afzelli* and purified Osp C from *B*. *garinii*. In the known positive panel, 58 of the 100 specimens were from individuals with a history of tick bite, erythema migrans (EM) or both. This may explain, in part, the poor sensitivity of the Trinity Biotech immunoblot in the known positive panel, despite the assay manufacturer’s claim in the package insert of 91% sensitivity in patients with EM.

The United States Centers for Disease Control and Prevention (CDC) has issued case definitions for Lyme disease for many years, the most recent being in 2017. In this case definition an IgG immunoblot is not considered definitively positive for surveillance and diagnosis unless reactivity is observed to five *B*. *burgdorferi* proteins, an approach recommended in 1995 that is still followed today [16]. We considered whether interpreting immunoblots using CDC criteria would be appropriate for this study. Differences in immunological responses to different strains of *B*. *burgdorferi* sl have been reported, with infected US individuals showing reactivity to a greater number of *B*. *burgdorferi* proteins than their European counterparts [31, 32]. Hence using CDC criteria in European patients resulted in reduced detection in well-characterised infected sera [31]. Given that only 60 of the 639 clinical specimens used in this study originated in North America, CDC criteria were not used to interpret immunoblot results.

Of the five immunoassays included in this study, three were included on the Australian Register of Therapeutic Goods (ARTG) at the time of writing (DiaSorin LIAISON CLIA, Euroimmun ELISA and NovaTec NovaLisa ELISA). This means that these assays and their manufacturers had fulfilled the regulatory requirements of Australia’s regulatory body, the Therapeutic Goods Administration. Only one of the immunoblots (Euroimmun Euroline) was on the ARTG. While the specificity of the Euroimmun Euroline immunoblot was estimated as 91% or 95% (presumed or known negative specimens respectively), using it to confirm reactive results from the DiaSorin LIAISON CLIA may give a false positive overall result. In the presumed negative specimen panel (n = 405), 23 specimens were falsely reactive on the DiaSorin Liaison CLIA. Of these, five were also positive on the Euroimmun Euroline immunoblot. Hence there is the possibility of reporting false positive results even when a confirmatory immunoblot is included in the algorithm. The lack of *B*.*burgdorferi* immunoblots on the ARTG is a challenge for Australian laboratories testing for Lyme disease.

There were limitations to this study that should be acknowledged. First we used archived specimens and allocated presumed positive and negative status by consensus results between assays; then we judged assays’ performances based on the consensus. This may have artificially enhanced or detracted from assay performances and may have affected some assays more than others. However, in the absence of a gold standard, we considered this approach was justifiable. Also having known positive and negative specimen panels in which the status was allocated to the specimens based on specific clinical, and serological characteristics offset the judgements based on presumed status. Second, specimens were contributed by participating laboratories based on their test results, which had been generated in many cases in the assays included in the study. This had the potential to bias the specimens that were included, especially when the numbers of specimens contributed by the laboratories were different from each other. We saw the effect of this with respect to the performance of the Immunetics C6ELISA in the presumed negative specimen panel, where the inclusion of a particular group of specimens biased the calculation of the assay’s negative agreement. Finally, we may have been able to draw further conclusions on assay performance if we had known definitively the relationship between timing of specimen collection and onset and / or type of symptoms where relevant.

We found that when using the same assay, discordance between study and clinical laboratories’ results occurred less than 2% of the time and that the assays agreed on positive results approximately 80% of the time and on negative results approximately 90% of the time.

These findings suggest that discordance in results between laboratories is most likely due to variation in algorithms or in different assays’ sensitivities or specificities rather than different results being reported from the same assay between laboratories.
